# Is Chemoembolisation of Value in Inoperable Primary Hepatocellular Carcinoma

**DOI:** 10.1155/1998/59271

**Published:** 1998

**Authors:** Jean-Luc Raoul

**Affiliations:** Centre E Marquis BP 6279 Rennes Cedex 35062 France

## Abstract

primary treatment for unresectable hepatocellular carcinoma (HCC). In this unit, 185 patients with a new diagnosis of HCC not amenable to surgery were seen between 1988 and 1991. Intended therapy for these patients was chemoembolisation with doxorubicin (60 mg/m^2^) and lipiodol, repeated at six week intervals until it was technically no longer possible o.r until complete tumour response had been obtained. Chemoembolisation was possible in 67 of the 185 (37%). Reasons for exclusion were portal vein occlusion (*n*=36), decompensated cirrhosis (n 44), distant metastases (*n*=5), diffuse tumour or unsuitable anatomy (tumour or vasculature) (*n*=11), patient refusal (*n*=11), and other (*n*=11). Patients excluded from treatment survived for a median of 10 weeks (range 3 days-19 months). In patients treated, 18 had small HCC (4cm) and 49 had large or multifocal HCC. Chemoembolisation was carried out a median of two sessions for small and three sessions for large tumours. Ten of 18 patients with small HCC showed a 50% or greater reduction in tumour size. Five of 49 patients with large or multifocal tumours showed a response to treatment. Median overall survival for treated patients was 36 weeks (range 3 days–4 years). One patient has subsequently undergone liver transplantation with no recurrence and minimal residual disease at transplantation. Two other patients are alive three years after chemoembolisation, one with no evidence of recurrent disease. No patient was thought suitable for surgery after their response to chemoembolisation. Chemotherapy related complications were seen in 22%. Complications were significantly more common in patients with larger tumours and poor liver reserve. Five patients died as a result of chemotherapy related complications. In conclusion, only one third of UK patients with unresectable HCC are treatable by chemoembolisation. Results with small tumours are encouraging, with a high response rate and the possibility of surgical intervention in previously inoperable disease. Large tumours, however, show a poor response and significant incidence of side effects, suggesting that this treatment offers little benefit in advanced disease.


required percutaneous drainage – none of them were infected whereas 3 of the drain group collections were infected.

The paper concludes that drainage after liver resection is unnecessary. I have several problems with this:

1. The power of this study to detect adverse outcome in the no drain group is very limited – with approximately 50 patients in each group, if a complication which occured with a rate of 5% in the drained group was three times more frequent in the undrained group this would *not* be detected.
2. The study does not show any advantage to not draining. In fact, there was significantly higher need for percutaneous post-op drainage.
3. Whilst two complications of drainage were seen – drain site abscess and drain site cancer recurrence, ie. the former would seem a relatively minor complication and even if the drain site appeared to be the only site of recurrence in the second patient, it would seem likely that if enough viable tumour cells were transplanted into the drain site that they would have developed at other sites in time.

David L. Morris
Professor of Surgery
Department of Surgery
The St George Hospital
Sydney NSW 2217 – Australia

### Is Chemoembolisation of Value in Inoperable Primary Hepatocellular Carcinoma

### ABSTRACT

*Ryder, S. D., Rizzi, P. M., Metivier, E., Karani, J. and Williams, R. (1996) Chemoembolisation with lipiodol and doxorubicin: applicability in British patients with hepatocellular carcinoma, Gut, 38, 125–128.*

**Chemoembolisation has been extensively used as primary treatment for unresectable hepatocellular carcinoma (HCC). In this unit, 185 patients with a new diagnosis of HCC not amenable to surgery were seen between 1988 and 1991. Intended therapy for these patients was chemoembolisation with doxorubicin (60 mg/m$^2$) and lipiodol, repeated at six week intervals until it was technically no longer possible or until complete tumour response had been obtained. Chemoembolisation was possible in 67 of the 185 (37%). Reasons for exclusion were portal vein occlusion (*n* = 36), decompensated cirrhosis (*n* = 44), distant metastases (*n* = 5), diffuse tumour or unsuitable anatomy (tumour or vasculature) (*n* = 11), patient refusal (*n* = 11), and other (*n* = 11). Patients excluded from treatment survived for a median of 10 weeks (range 3 days-19 months). In patients treated, 18 had small HCC ( 4 cm) and 49 had large or multifocal HCC. Chemoembolisation was carried out a median of two sessions for small and three sessions for large tumours. Ten of 18 patients with small HCC showed a 50% or greater reduction in tumour size. Five of 49 patients with large or multifocal tumours showed a response to treatment. Median overall survival for treated patients was 36 weeks (range 3 days–4 years). One patient has subsequently undergone liver transplantation with no recurrence and minimal residual disease at transplantation. Two other patients are alive three years after chemoembolisation, one with no evidence of recurrent disease. No patient was thought suitable for surgery after their response to chemoembolisation. Chemotherapy related complications were seen in 22%. Complications were significantly more common in patients with larger tumours and poor liver reserve. Five patients died as a result of chemotherapy related complications. In conclusion, only one third of UK patients with unresectable HCC are treatable by chemoembolisation. Results with small**

**tumours are encouraging, with a high response rate and the possibility of surgical intervention in previously inoperable disease. Large tumours, however, show a poor response and significant incidence of side effects, suggesting that this treatment offers little benefit in advanced disease.**

*Keywords:* Chemoembolisation, lipiodol, hepatocellular carcinoma, doxorubicin

### PAPER DISCUSSION

Only a few years ago, surgery was the only possibility for patients with hepatocellular carcinoma. Today, with the large number of therapeutic approaches being proposed, including some with promising preliminary phase II results, it could be said that the opposite is true, too many possibilities for too few patients [1].

One of the main avenues of research has been the locoregional approach using Lipiodol [2] as a vector to deliver either a chemical agent or irradiation directly into the tumour site, increasing the antitumoural activity by embolism-induced transitory ischaemia. The first trials using chemoembolisation led to much enthusiasm, which was abated when two phase III trials [3, 4] were unable to demonstrate any benefit in survival over therapeutic abstention. Moreover, in addition to negative results, a recently published multicentric trial [4] also demonstrated the limitations on the feasibility of chemoembolisation since among the 778 patients seen for hepatocellular carcinoma in the 24 participating centres over a 30 month period, only 96, *i.e.* 12%, could be included in the study. The other patients were excluded because of liver failure (30%), vascular contraindications (22%), indications for surgery (17%) or alcoholisation (2%), extrahepatic metastases (6%) and patient refusal (14%).

Ryder *et al.* report chemoembolisation data obtained in a population of British patients. These authors saw 195 patients in their unit over a 4-year period. Surgery was proposed as first intention therapy in 10 (5%). Despite very wide inclusion criteria, only 67 (36%) of the non-operated patients were treated with chemoembolism; liver failure and portal thrombosis being the main exclusion factors. The wide inclusion criteria probably explain the high rate of treatment-related side effects: 22% of the cases with 5 deaths (8% of the treated patients). In our unit, we have observed comparable results; morbidity and mortality depend greatly on the severity of the underlying liver disease. Ryder's group clearly demonstrate this fact since complications occurred in 31% of their patients in class C in the Child classification compared with 10% in class A patients. These authors also note a clear relationship between tumour size and frequency of iatrogenic accidents described in 57% of the patients with a tumour volume greater than 268 $cm^3$ (diameter = 8 cm). Using response rate as a measurement, efficacy was low, hardly 20%; it was somewhat better in small tumours (56%) and very low for multifocal or voluminous tumours (10%). This relationship between tumour size and response is well-known and logical. Vascularization is generally better in small tumours than in larger, often necrotic, tumours. The intensity of Lipiodol retention, or more precisely of the therapeutic agent it vehicles, is directly correlated with tumour size [5]. When a response is obtained, it usually occurs rapidly after 1 or 2 sessions. These authors did not obtain any response if none was evidenced after the second injection. No beneficial effect of treatment on survival could be evidenced in this non-randomised trial. Nevertheless, the authors do report 4 patients who survived more than 3 years including 1 who later had liver transplantation after a complete 6-month response to the first embolisation.

Needless to say, this very interesting trial confirms the dead-end situation we are in for treatment of patients with hepatocellular carcinoma. One could criticise the methodolody of this trial since the chemolipiodolisation technique probably allowed exessively rapid dexorubicin release so that the effect was similar to a single embolisation [6], but without going into the discussion on the efficacy of the chemoembolisation protocol, it can be noted that the technique could only be used in about one-third of the potential cases. In addition, the number of patients who could be expected to benefit from chemoembolisation without too great a risk – patients with small tumours and low-grade cirrhosis – was small, less than 10 to 15% of the potential cases.

Consequently, although we have an apparently impressive armamentarium of therapeutic options (resection, transplantation, alcoholisation, chemoembolism), in clinical practice we can propose them to only a very few patients. For the large majority of our patients with hepatocellular carcinoma, we have little to offer.

Further options will undoubtedly be developed in the future, and perhaps more importantly, better indications for optimal application of those already in use will be defined. Theoretically, transplantation, resection, alcoholisation, and perhaps chemoembolisation, might be beneficial for patients with small tumours and low-grade cirrhosis. To identify these patients, we urgently need randomised trials comparing resection and transplantation, resection and alcoholisation or even resection and chemoembolisation. For patients who have a multifocal diffuse tumour or a tumour developing in a severely cirrhotic liver, *i.e.* approximately 50% of our patients, it will be important to evaluate the eect of less aggressive treatments such as antihormone therapy or intra-arterial injections of radioactive Lipiodol. This latter technique, resulting in one randomised study in a better survival than best supportive care achieved in patients with thrombosis of the portal vein [7], does not have the disadvantages of chemoembolisation but has the same efficacy. For the time being, it is unlikely that any treatment should be proposed for patients with advanced-stage cirrhosis, whatever the size of the tumour, since the fatal course is determined more by cirrhosis than by cancer.

With this paper, more than a decade of non-randomised trials and unfortunately unfounded enthusiasm draws to an end. The work reported has clearly demonstrated the limitations of chemoembolisation, emphasising the moderate feasibility of the technique, its frequent and severe side effects, and its rather disappointing results. The reported data will help describe several very different subpopulations who could benefit from different treatments and point to the urgent need for therapeutic trials to identify the most appropriate technique in these subpopulations.

Jean-Luc Raoul, MD, PhD
Centre E Marquis, BP 6279
35062 Rennes Cedex-France

### *References*

[1] Venook, A. P. (1994). Treatment of hepatocellular carcinoma: too many options? *J Clin Oncol.*, **12**, 1323 – 34.
[2] Raoul, J. L., Bourguet, P., Bretagne, J. F., Duvauferrier, R., Coornaert, S., Darnault, P. *et al.* (1998). Hepatic artery injection of I-131-labeled Lipiodol. Part I. Biodistribution study in patients with hepatocellular carcinoma and liver metastases. *Radiology*, **168**, 541 – 5.
[3] Pelletier, G., Roche, A., Ink, O., Anciaux, M. L., Derhy, S., Rougier, P. *et al.* (1990). A randomized trial of hepatic arterial chemoembolization in patients with unresectable hepatocellular carcinoma. *J Hepatol.*, **11**, 181 – 4.
[4] Groupe, d'Etude et de Traitement du Carcinome Hépatocellulaire. (1995). A comparison of Lipiodol chemoembolization and conservative treatment for unresectable hepatocellular carcinoma. *N Engl J Med.*, **332**, 1256 – 61.
[5] Maki, S., Konno, T. and Maeda, H. (1985). Image enhancement in computerized tomography for sensitive diagnosis of liver cancer and semiquantification of tumor selective drug targeting with oily contrast medium. *Cancer*, **56**, 751 – 7.
[6] Heresbach, D., Raoul, J. L., Bentue-Ferrer, D., Bretagne, J. F., Van den Driessche, J. and Gastard, J. (1989). Chimiothérapie couplée au Lipiodol. Etude in vitro de la cinétique de libération de ladriamycine. *Gastroentérol Clin Biol.*, **13**, 775 – 8.
[7] Raoul, J. L., Guyader, D., Bretagne, J. F., Duvauferrier, R., Bourguet, P., Bekhechi, D. *et al.* (1994). Randomized controlled trial for hepatocellular carcinoma with portal vein thrombosis: intra-arterial iodine-131-iodized oil versus medical support. *J Nucl Med.*, **35**, 1782 – 7.

